# Transcriptional Regulator PerA Influences Biofilm-Associated, Platelet Binding, and Metabolic Gene Expression in *Enterococcus faecalis*

**DOI:** 10.1371/journal.pone.0034398

**Published:** 2012-04-04

**Authors:** Scott M. Maddox, Phillip S. Coburn, Nathan Shankar, Tyrrell Conway

**Affiliations:** 1 Advanced Center for Genome Technology, University of Oklahoma, Norman, Oklahoma, United States of America; 2 Department of Pharmaceutical Sciences, The University of Oklahoma Health Sciences Center, Oklahoma City, Oklahoma, United States of America; University of South Dakota, United States of America

## Abstract

*Enterococcus faecalis* is an opportunistic pathogen and a leading cause of nosocomial infections, traits facilitated by the ability to quickly acquire and transfer virulence determinants. A 150 kb pathogenicity island (PAI) comprised of genes contributing to virulence is found in many enterococcal isolates and is known to undergo horizontal transfer. We have shown that the PAI-encoded transcriptional regulator PerA contributes to pathogenicity in the mouse peritonitis infection model. In this study, we used whole-genome microarrays to determine the PerA regulon. The PerA regulon is extensive, as transcriptional analysis showed 151 differentially regulated genes. Our findings reveal that PerA coordinately regulates genes important for metabolism, amino acid degradation, and pathogenicity. Further transcriptional analysis revealed that PerA is influenced by bicarbonate. Additionally, PerA influences the ability of *E. faecalis* to bind to human platelets. Our results suggest that PerA is a global transcriptional regulator that coordinately regulates genes responsible for enterococcal pathogenicity.

## Introduction

As a commensal member of the intestinal microbiota, the enterococci play an important role in establishing a healthy GI tract and typically coexist in the host as a relatively small, yet stable, population. Alternatively if the delicately balanced host/commensal relationship is disrupted, if specific environmental cues are detected, or if virulence traits are acquired, enterococci can act as opportunist pathogens capable of multiple-site infections, including infections of the heart, urinary tract, and bloodstream [1], [2], [3]. In an effort to better understand the differences between commensal and pathogenic enterococci, studies of pathogenic enterococci increasingly seek to discover which traits promote virulence, how these traits are inherited and what mechanisms are used to coordinately regulate these traits to achieve pathogenicity.

While the enterococci have been known as infective agents for more than 100 years [4], the majority of information regarding the acquisition and deployment of virulence traits has been gathered in the last few decades [5], [6], [7]. As a result of these studies, we have a clearer picture of how the enterococci successfully transition from a commensal to a pathogen. At the heart of this transition is enterococcal promiscuity: the ease and frequency with which many strains acquire and transmit mobile genetic elements harboring loci that contribute to pathogenesis. In addition to being intrinsically resistant to a broad range of antimicrobial agents, enterococci have evolved resistance to many antibiotics by acquiring plasmids or transposons comprised of genes that confer resistance. Developing antibiotic resistance has increased the pathogenic potential of the enterococci, as is evident by these organisms becoming the leading cause of surgical site infections, the second leading cause of bloodstream infections and the third leading cause of nosocomial urinary tract infections [8]. Furthermore, antibiotic resistant strains are more likely to contain mobile genetic elements that may harbor virulence traits [9]. Especially problematic are strains that acquire both antibiotic resistance and virulence traits, as the concurrence of these factors is correlated with strains capable of producing infection outbreaks on a global scale [10].

Facilitating the spread of virulence traits in a particularly efficient manner are pathogenicity islands (PAI). PAI's are characterized as clusters of genes encoding proteins with roles involving transfer functions, virulence, stress survival, and transcriptional regulation [11]. Furthermore these mobile genetic elements can be distinguished from the native chromosome by a significantly different G+C content [11]. While first discovered in pathogenic *Escherichia coli* [12], [13], these mobile genetic elements are disseminated throughout many bacterial genera [11]. A 153 kb PAI consisting of 129 open reading frames was discovered in *Enterococcus faecalis* MMH594 and shown to disperse to many *E. faecalis* strains of various origins [10], [14], [15]. This PAI contains many loci with roles in virulence, including *esp* (encodes enterococcal surface protein), cytolysin toxin, and aggregation substance, as well as factors potentially involved in horizontal transfer and gastrointestinal tract colonization [14]. Esp is enriched among infection-derived isolates and has been shown to increase *in vitro* biofilm formation [16], [17]. The eight genes comprising the cytolysin operon (*cylR1*, *cylR2*, *cylL_L_*, *cylL_S_*, *cylMBAI*) form a two-peptide lytic toxin [18], [19]. Cytolysin toxin is effective against both prokaryotic and eukaryotic cells [20], [21], and contributes to mortality in various pathogenic models of infection [22], [23], [24]. A pheromone-inducible aggregation substance (AS) can also be found in many enterococcal strains. AS promotes aggregation and conjugation [25], [26], increases enterococcal adherence to and uptake in eukaryotic cells [27], [28] and increases bacterial survival inside the macrophage [29].

Frequently, PAI's contain genes encoding transcriptional regulators with various regulatory schemes, and the *E. faecalis* PAI is no exception [11], [14]. The *E. faecalis* PAI encodes an AraC-type regulator, named PerA (for pathogenicity island-encoded regulator) [14], [30]. PerA is enriched among clinical *E. faecalis* isolates and lies adjacent to the aforementioned PAI-encoded virulence traits, which suggests PerA-dependent regulation of these genes [14]. Through mutational analysis, we have previously shown that PerA influences biofilm formation in a medium-specific manner and contributes to virulence in a mouse peritonitis model [30]. Additionally, the PerA-deficient strain was significantly attenuated during macrophage survival, further supporting the role of PerA as an important regulator of *E. faecalis* pathogenesis [30].

Prompted by the observation that PerA coordinates *E. faecalis* virulence in the mouse peritonitis infection model, we sought to identify the genes that are regulated directly or indirectly by PerA. We used Affymetrix GeneChip microarrays to experimentally define the PerA regulon throughout exponential growth, upon transition into stationary phase and during stationary phase persistence. Our results suggest that PerA primarily regulates genes located outside of the PAI in a growth phase-dependent manner. These PerA-regulated genes are located throughout the *E. faecalis* chromosome and include loci responsible for amino acid metabolism, biofilm formation and phage-associated genes putatively involved in platelet binding. Further experimentation revealed that PerA influences the ability of *E. faecalis* to bind human platelets and respond to the presence of bicarbonate. Taken together with our previous findings [30], we interpret these results to mean that PerA acts as a global transcriptional regulator to coordinately regulate genes responsible for enterococcal pathogenicity.

## Results

### Overview of microarray data

**Figure 1:**
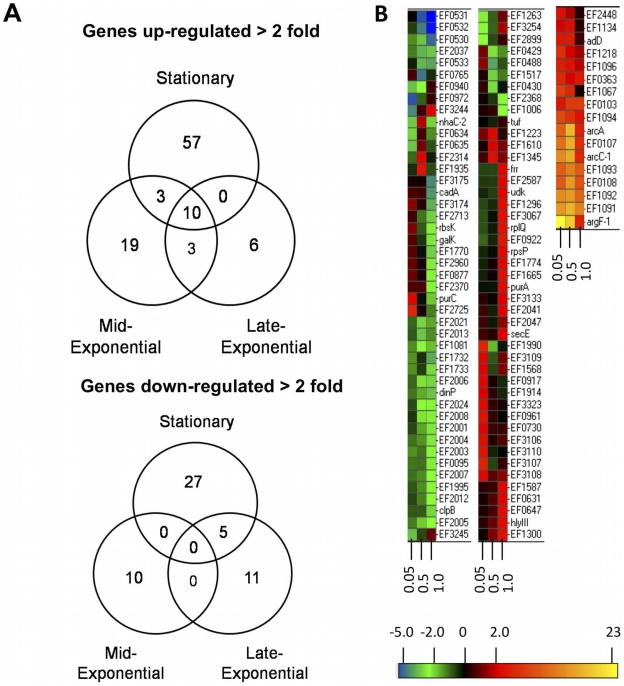
Comparisons of microarray results for E99 and DBS01. Control RNA was extracted from E99 and used to normalize the test RNA extracted from DBS01 (DBS01 : E99). All data presented here are shown as fold change in gene expression (test : control). (A) Upper diagram: Venn diagram comparing significantly up-regulated genes (>2 fold) in DBS01 during mid-exponential, late-exponential and stationary phase. Lower diagram: Venn diagram comparing significantly down-regulated genes (>2 fold) in DBS01 during mid-exponential, late-exponential, and stationary phase. (B) Hierarchically-clustered heat map of all genes differentially regulated>twofold between DBS01 and E99.

PerA is an AraC-type transcriptional regulator that contributes to pathogenesis in *E. faecalis* [30]. To define the PerA regulon, transcriptional profiling was performed on *E. faecalis* E99 and an isogenic Δ*perA* mutant strain (designated DBS01) using RNA extracted from both strains at time points corresponding to mid-exponential, late-exponential, and stationary phase (O.D. 600 nm∼0.05, 0.5, and 1.0, respectively). The RNA was reverse-transcribed and subsequently hybridized to *E. faecalis* V583 genome microarrays. All array data shown are expressed as ratios (DBS01 : E99) and considered to be significant if gene expression was induced or repressed in the mutant strain greater than twofold. The PerA regulon is extensive, as transcriptional analysis revealed 151 genes differentially regulated>twofold (log_2_ = 1) in DBS01 (Table S1). Of these 151 genes, 98 were up-regulated and 53 were down-regulated. Nearly one-third (46 of 151) of the differentially regulated genes have unknown function, 20 are involved in metabolic functions, and 19 encode transport-related genes. Of the 98 up-regulated genes, 19 are up-regulated in mid-exponential phase only, 6 are up-regulated in late-exponential phase only, and 57 are up-regulated only in stationary phase (Figs. 1A and 1B). Of the 53 down-regulated genes 10 are down-regulated only in mid-exponential phase, 11 are down-regulated only in late-exponential phase, and 27 are down-regulated only in stationary phase (Fig. 1A). These data suggest that while PerA is primarily a negative regulator, it can also act as a dual regulator, as a positive influence on gene expression is also noted (Figs. 1A and 1B). Additionally, the PerA target genes show a high degree of growth-phase dependent regulation, with the highest degree of influence occurring in stationary phase (Figs. 1A and 1B). Finally, we verified expression of *perA* in E99 using quantitative reverse transcription PCR (qRT-PCR) and found that expression of *perA* was maximal during stationary phase (data not shown); a finding that reflects the high degree of PerA regulation at this time point.

### DBS01 shows altered expression of PAI-related genes

**Figure 2:**
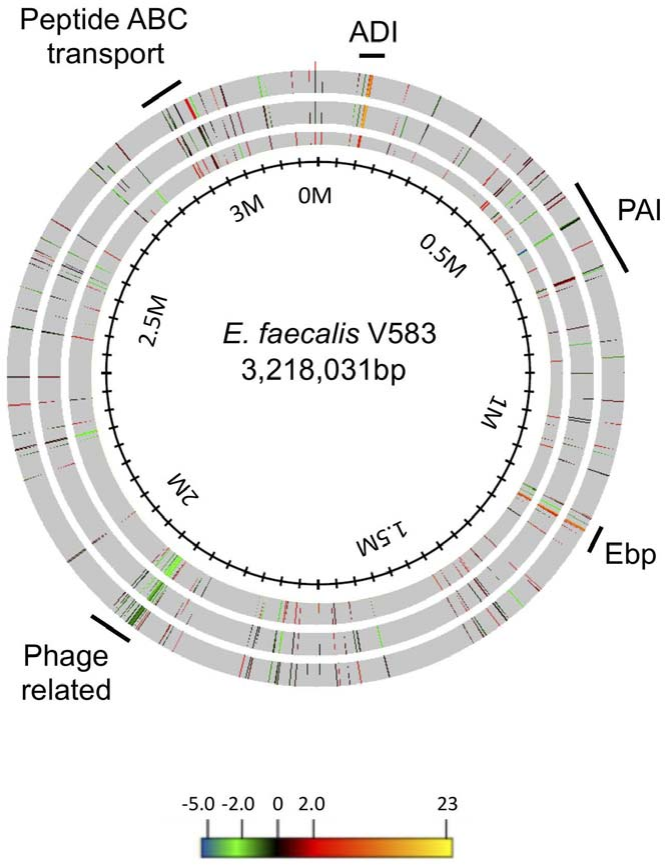
All genes differently regulated in DBS01 mapped onto the *E. faecalis* chromosome. The outer ring displays those genes differentially regulated during mid-exponential phase. The middle ring displays those genes differentially regulated during late-exponential phase. The inner ring displays those genes differentially regulated during stationary phase. The innermost circle displays the location relative to position zero in millions of base pairs of the *E. faecalis* V583 genome. The locations of the arginine deiminase (ADI) and enterococcal biofilm-associated pilus (Ebp) operons, the *E. faecalis* pathogenicity island (PAI), and a phage related element are indicated.

The 153 kb PAI carries virulence determinants (including cytolysin, Esp, and aggregation substance) adjacent to *perA* [14], [30]. The proximity of the *perA* gene to genes with ascribed roles in virulence is suggestive of PerA regulation of PAI genes. In DBS01, 5 PAI genes were differentially regulated in any of the time points studied (Figs. 1B and 2). During mid-exponential growth the EF0579 gene was induced (Fig. 1B). This locus encodes a putative TetR-family protein with unknown function in *E. faecalis*. Four genes encoding hypothetical proteins (EF0488, EF0531, EF0532, EF0533) were down-regulated between 2 and 4 fold in DBS01 at late-exponential phase (Fig. 1B). In stationary phase the EF0579 gene was again induced, while the EF0488 gene was no longer differentially regulated (Fig. 1B). The microarrays used in this study were developed using the strain V583 sequenced genome. V583 is missing portions of the cytolysin operon, *nsr* and *gls24*-like genes, and the entire *esp* gene due to a spontaneous 17 kb deletion within the PAI [14]. Therefore, qRT-PCR was used to determine the expression of these PAI genes found in strain E99 but absent in V583. qRT-PCR revealed no differential regulation of these genes in DBS01 at any time point tested (data not shown). The differential regulation of PAI hypothetical genes, but not genes with previously ascribed roles in virulence, may indicate PerA-dependent control of genes with an unknown function in enterococcal pathogenicity; however this possibility remains to be studied.

### The transcription of many housekeeping genes is altered in DBS01

AraC-type regulators are known to control a variety of cellular processes, including metabolism and other housekeeping functions [31]. We mined the transcriptome to determine if any housekeeping genes were regulated by PerA, and found a number of genes differentially expressed in DBS01. A number of genes involved in basic cellular metabolism were down-regulated in DBS01, including *galK*, *rbsK* (EF2961) and *rbsD* (EF2960) (Fig. 1B). *galK* encodes for galactokinase, while *rbsK* and *rbsD* encode for ribokinase and a ribose transporter, respectively, and are potentially required for transport and metabolism of galactose and ribose. Many housekeeping genes are induced in DBS01, including genes encoding ribosomal proteins (*rplQ*, *rpsP*, *rpsD* [EF3070], *rpmB* [EF3116] and *rpmH* [EF3333]) and pyrimidine nucleotide biosynthetic genes (*purA*, EF0014) (Fig. 1B). Lastly, putative peptide ATP-binding cassette (ABC) transporters were significantly induced in DBS01. While poorly studied in *E. faecalis*, these peptide transporters generally provide nutrients to bacteria in the form of amino acids or short peptides [32], [33]

### PerA regulates biofilm-related genes in E99

**Figure 3:**
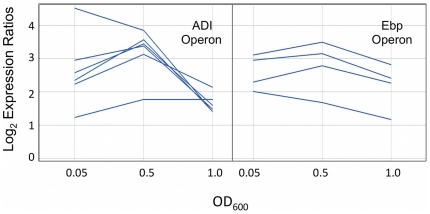
Plots comparing the log_2_ expression ratios of the arginine deiminase (ADI) and enterococcal biofilm associated pilus (Ebp) operons in DBS01.

*E. faecalis* E99 is a urinary-tract isolate possessing a high biofilm phenotype [34]. Recently a ubiquitous enterococcal locus was characterized and named *ebp* [35]. The *ebpABC* operon encodes the enterococcal biofilm-associated pilus and contributes to endocarditis, urinary tract infections (UTI), and biofilm formation [35], [36]. The EbpABC proteins are polymerized through the activity of Bps (formerly, SrtC), and together are required for maximal biofilm production in *E. faecalis* [35]. EbpR acts as a transcriptional activator of *ebpABC* and positively influences biofilm formation [37]. As previously shown, the PerA regulator influences E99 biofilm formation in a medium-dependent manner [30]. To determine if PerA regulates *ebpABC* and *bps* gene expression, we compared the transcriptome of DBS01 to E99 during mid-exponential, late-exponential, and stationary phase. In DBS01, the *ebpABC* operon and associated *bps* gene was induced between 4 and 8-fold during mid-exponential and stationary phases (average operon induction = 6.2-fold) (Fig. 3). The transition from mid-exponential to late-exponential growth was concomitant with an increase in expression of the *ebpABC* operon (average operon induction = 8.8-fold) (Fig. 3). Induction of the *ebpABC* and *bps* genes was confirmed by using qRT-PCR (Table 2). The high degree of *ebpABC* up-regulation shown here, as well as the increase in biofilm formation previously shown in DBS01 [30], suggests that PerA may act as a repressor of the *ebpABC* operon and associated *bps* gene in E99.

Next we sought to examine if PerA regulates other biofilm-related genes found in E99, including *esp*, the *bee* locus, and *fsrABCD* operon. *esp* encodes for enterococcal surface protein, a high-molecular weight protein that has been shown to enhance biofilm formation [16], [38]. The *bee* locus is a unique five-gene system that contributes to the high biofilm phenotype found in E99 [34]. The microarrays used for this experiment were derived from the *E. faecalis* V583 sequenced genome. V583 is missing the *esp* gene due to a 17 kb PAI deletion [14], and does not contain the conjugative plasmid harboring the *bee* locus (unpublished results). Therefore it was impossible to examine gene expression of these by using microarrays. qRT-PCR was used to determine possible changes in gene expression for the *esp* and *bee* loci. When comparing DBS01 and E99 using qRT-PCR, no significant differential regulation of the *esp* or *bee* loci in any of the three growth phases tested was observed (data not shown).

The *fsr* system, encoded by the *fsrABCD* operon, is similar to the *agrABCD* operon found in *Staphylococcus aureus* [39]. Through the production of gelatinase-biosynthesis activating pheromone, *fsr* activates two genes encoding a gelatinase (*gelE*) and a serine protease (*sprE*) resulting in biofilm formation [39], [40], [41], [42], [43]. Though little is known about the *fsr* or *gelE-sprE* loci in E99, approximately 60% of *E. faecalis* clinical isolates produce gelatinase [44]. We searched the microarray data and found no differentially regulated genes in either the *fsr* or *gelE-sprE* loci in DBS01. Taken together these data suggest that PerA may act to repress the *ebpABC* operon and associated sortase while having little to no influence on the expression of the *esp*, *bee* or *fsr* loci under the conditions tested.

### *perA* and *ebpABC* respond to the presence of bicarbonate in E99

**Figure 4:**
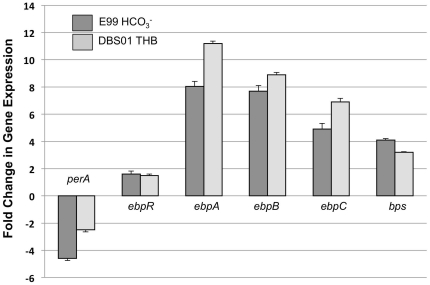
*perA, ebpR-ebpABC* and *bps* gene expression in E99 grown in THB supplemented with 100 mM sodium bicarbonate (dark bars) or DBS01 grown in THB (light bars). Microarray assays were performed twice. The values shown are mean expression intensities (mean ± SD) of biological replicates. Fold changes in gene expression were calculated by comparing E99 grown in THB to E99 grown in THB+100 mM sodium bicarbonate and by comparing DBS01 grown in THB to E99 grown in THB.

Using β-gal assays and qRT-PCR, Bourgogne et al. have recently shown that *E. faecalis* OG1RF *ebpABC* expression increases when grown in sodium bicarbonate in an *ebpR*-dependent manner [45]. Our data suggest that PerA acts as a repressor of the *ebpABC* locus (Figs. 1B and 3). Furthermore, AraC-type regulators are known to respond to bicarbonate, including RegA in *Citrobacter rodentium* and ToxT in *Vibrio cholerae* [46], [47]. Given that OG1RF lacks the *E. faecalis* PAI, including *perA*, we were curious to determine the effects of bicarbonate on *ebpABC* expression in E99. To do this we analyzed the transcriptome of E99 grown in THB supplemented with 100 mM sodium bicarbonate. When compared to E99 grown in THB, *perA* expression was down-regulated in the presence of bicarbonate while *ebpR* (the activator of *ebpABC*) was moderately induced (Fig. 4). Furthermore, the average *ebpABC* expression increased approximately 7-fold (*ebpA* = 8.0, *ebpB* = 7.7, *ebpC* = 4.9), with the biofilm and pilus-associated sortase (*bps*) being induced 4-fold (Fig. 4).

We reasoned that if PerA represses the *ebpABC* locus, a down-regulation of *perA* in the presence of bicarbonate would cause a response similar to that seen in DBS01 (Δ*perA*). When comparing the transcriptome of E99 grown in THB supplemented with 100 mM sodium bicarbonate to DBS01 grown in THB, similar trends in *perA*, *ebpR-ebpABC* and *bps* gene expression are observed (Fig. 4). These results suggest that *perA* is down-regulated in the presence of bicarbonate, concomitant with an induction of the *ebpR-ebpABC* and *bps* loci.

### Effect of the *perA* mutation on expression of ADI pathway

The arginine deiminase (ADI) system is used by many microorganisms to generate ATP via arginine fermentation [48]. Genes comprising the ADI pathway in *E. faecalis* are arranged as the *arcABCRD* operon (ArcA, arginine deiminase, ArcB, ornithine carbamolytransferase; ArcC, carbamate kinase; ArcR, Crp/Fnr regulator, ArcD arginine/ornithine antiporter), and are known to be transcribed in the presence of arginine [49]. The ADI operon has a complex regulatory scheme with binding sites for two arginine-sensitive regulators (ArgR1 and ArgR2), a catabolite control protein (CcpA), as well as a protein involved in *E. faecalis* pathogenicity (Ers) [49], [50]. The regulatory roles of ArgR1 and ArgR2 remain unclear, however multiple Arg boxes can be found upstream of the ADI operon and data suggests that ArgR2 may act as an arginine-specific signal transducer [49]. Furthermore, expression of *argR1* and *argR2* increases in the presence of arginine and is absent in glucose containing medium [49]. In DBS01 the *arcABCRD* operon is highly up-regulated in all time points tested (Fig. 3). On average, the *arcABCRD* operon is induced 7.6-fold during mid-exponential growth and plateaus upon entrance into late exponential phase induced 11-fold. The average expression of the *arcABCRD* genes is up-regulated 3-fold during stationary phase. This pattern of ADI pathway regulation is similar to that previously observed in *E. faecalis*. Bourgogne et al. found that the enterococcal FsrB transcriptional regulator negatively influences *arcABC* expression during transition from exponential to stationary phase; though it is unclear if this regulation is direct or indirect [51]. Riboulet-Bisson et al. have shown that the Ers regulator activates *arcABC* expression by binding upstream of the *arcA* gene [50]. For unknown reasons and in contrast to this study, *arcRD* gene expression was not differentially regulated by FsrB or Ers [50], [51]. In DBS01 *argR1* gene expression was induced at all time points tested (Fig. 3) while the *argR2* gene was not differentially regulated (data not shown). The *argR1* and *arcABCRD* genes account for 60% (6 out of 10) of the genes up-regulated in all time points tested (Figs. 1A and 3), suggesting the PerA regulator may act as a repressor of arginine catabolism in *E. faecalis*.

### PerA regulates a putative temperate bacteriophage in E99

**Figure 5:**
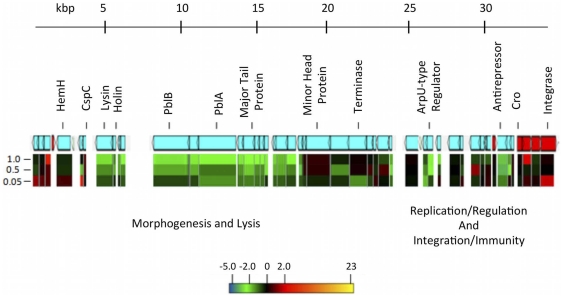
Map of *E. faecalis* V583 phage 04. The putative proteins were compiled using the annotated V583 sequence. The direction of transcription is shown in blue (reverse) and red (forward). Heat maps of expression ratios (fold change) for DBS01 are shown for mid-exponential (O.D. 600 = 0.05), late-exponential (O.D. 600 = 0.5) and stationary phase (O.D. 600 = 1.0).

Temperate bacteriophages are disseminated throughout many gram-positive bacteria, including *E. faecalis*. The *E. faecalis* V583 sequenced genome contains seven regions arising from integrated phages [52]. Though the role of these phages in *E. faecalis* virulence has yet to be discovered, each of these mobile elements contains homologs of virulence determinants from *Streptococcus mitis* phage SM1 [52], [53]. We mined the microarray data for each of these putative phage-related genes, and found a cluster of genes similar to phage 04 in V583 that was differentially regulated in DBS01 (Figs. 2 and 5). This element spans *ef1985–ef2043* and contains putative replication, integration and virulence functions. The majority of genes on the phage display either no change or non-significant induction or repression in DBS01 throughout all growth phases. However a group of genes show significant growth phase-independent repression in DBS01, including homologs of *pblA*, *pblB* and a gene encoding a putative lysin (Fig. 5). PblA and PblB mediate bacterial attachment to platelets in *S. mitis* [54]. The lysin protein serves a dual purpose: permeablizing the bacterial cell wall, thus permitting release of PblA and PblB, and binding to platelets through interaction with fibrinogen and fibrinogen receptors [55], [56]. *E. faecalis* is known to aggregate human platelets, yet the molecular mechanisms coordinating this process have not been discovered [57]. The repression of *pblA*, *pblB* and lysin in DBS01 suggests that PerA influences the expression of genes putatively involved in platelet binding and cell wall permeability residing on a temperature bacteriophage in E99.

### PerA influences the binding to human platelets

**Figure 6:**
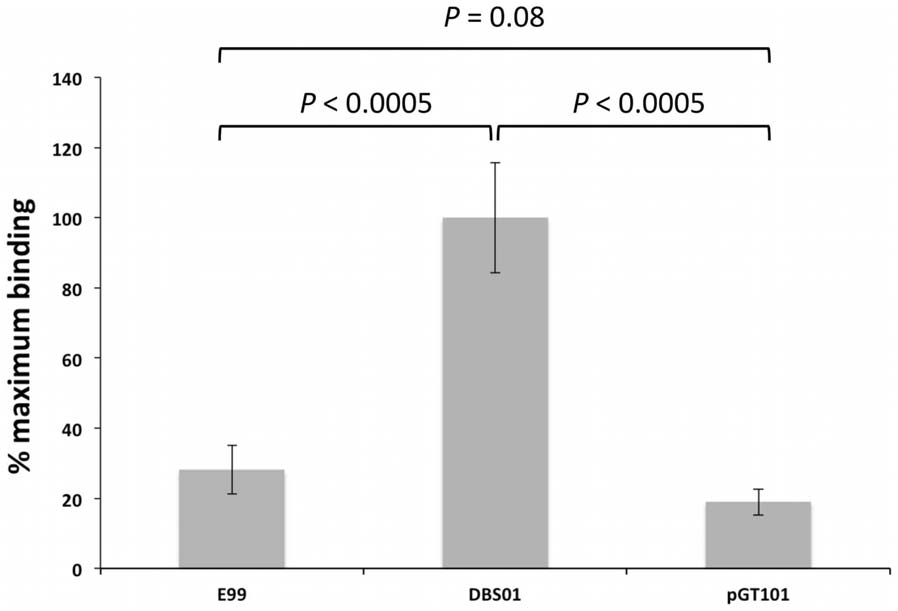
Platelet binding activity of E99 and DBS01. The values shown are percent of wild-type (E99) binding (mean ± SD). Differences in platelet binding efficiencies were determined using an unpaired *t*-test (P = 0.05). Platelet binding assays were performed in triplicate and each experiment was repeated thrice (n = 9).

PerA differentially regulates two distinct loci potentially important in bacterial attachment to human platelets. First are the putative *pblA*, *pblB* and lysin genes residing on a temperate bacteriophage. Next is the Ebp pilus, which has recently been shown to mediate bacterial attachment to human platelets [58]. Given that genes potentially involved in platelet binding were both induced and repressed in DBS01 (the *ebp* and phage-related loci, respectively), we sought to determine if DBS01 showed an altered ability to bind human platelets. To assess this we compared the ability of E99 and DBS01 to adhere to human platelets immobilized in microtiter plates. As shown in Fig. 6, DBS01 binds human platelets significantly (*P*<0.0005, unpaired *t*-test) better than the E99 wild-type strain. DBS01 bound platelets approximately 5-fold better than E99. When DBS01 contained a plasmid-encoded copy of *perA* (pGT101), platelet-binding abilities were restored to the wild-type levels (Fig. 6). These results suggest that the inactivation of *perA* increases platelet binding in DBS01, possibly through the derepression of the *ebpABC* locus.

## Discussion

The *perA* gene is located on the *E. faecalis* PAI, adjacent to loci with ascribed roles in virulence and genes with putative metabolic functions [14]. Given its location, it was our hypothesis that the primary function of PerA was to regulate the expression of PAI genes in *E. faecalis*. However, transcriptional analysis revealed that in DBS01 only 5 PAI genes of unknown function were altered in gene expression during the time course study. To our surprise the overwhelming majority of genes differentially regulated in the Δ*perA* mutant were chromosomally located yet not residing within the PAI. McBride et al. [10] have recently suggested that the enterococcal PAI is comprised of clusters of genes that likely undergo horizontal transfer as modules. Additionally, portions of the enterococcal PAI have been shown to conjugatively transfer both *in vitro* and *in vivo* [15]. These findings raise the possibility that PerA is able to transfer to strains lacking the PAI and subsequently exert alien control of native genes. In this scenario, the acquisition of the transcriptional regulator PerA could effect a rapid physiological change in the recipient. In *Salmonella*, HilD, a transcriptional regulator encoded on the *Salmonella* pathogenicity island SPI-1, has been shown to regulate genes on the evolutionary distinct SPI-2 pathogenicity island [59]. Furthermore, *E. coli* strain K12 genes can be regulated by Ler, a regulator located on the locus for enterocyte effacement (LEE) pathogenicity island of strain O157:H7 [60]. Our data suggest that PerA may have the ability to control native chromosomal genes upon entry into a recipient; however, the ability of PerA to transfer into an enterococcal strain lacking the PAI and regulate native genes remains to be tested.

Biofilm formation is often a key component of bacterial pathogenesis [61], [62], [63]. Though not necessarily a virulence trait, as biofilms are also produced by many avirulent bacteria, biofilms contribute to pathogenicity by increasing resistance to antibiotics and environmental stresses [64]. In *E. faecalis*, biofilms are correlated with infective endocarditis [37] and urinary tract infections [36], and promote bacterial survival inside phagocytes [65]. PerA has been shown to influence biofilm formation in a medium specific manner, as a *perA*-deficient strain designated DBS01 produced more biofilm than the WT strain E99 [30]. Transcriptional profiling revealed that the enterococcal biofilm associated pilus (*ebp*) locus, a ubiquitous determinant important for biofilm production [35], was up-regulated in DBS01. This makes possible the interesting scenario where the PAI-residing *perA* could transfer to recipient strains and influence biofilm formation through regulation of the *ebp* locus.

Expression of the *ebp* genes is controlled through multiple transcriptional regulators. In addition to the PerA-dependent repression of the *ebp* operon (Figs. 1B and 3), these genes are activated through the action of EbpR [37]. More recently Gao et al. have shown that expression of the *ebp* locus in *E. faecalis* OG1RF is influenced by *rnjB*, a gene encoding RNase J2 [66]. OG1RF strains deficient in RNase J2 expression have reduced *ebpABC* transcript levels and fail to produce Ebp pili, however the regulatory mechanism responsible for these observations is currently unknown [66]. Though poorly studied in *E. faecalis*, RNase J1 and J2 are highly conserved proteins encoded by *rnjA* and *rnjB*, respectively [66], [67]. In *B. subtilis*, the RNase J1 and J2 enzymes form heterotetramer complexes and are typically involved in mRNA processing, stability and turnover [68], [69]. We do not know if E99 possesses *rnjA* and *rnjB*, however these genes appear to be ubiquitous as every *E. faecalis* sequenced genome contain these loci [66]. Furthermore, we do not know how PerA, EbpR, and RNase J1 and J2 are structured within the regulatory network controlling Ebp pilus formation. It is possible that RNase J1 and J2 function independently of either PerA or EbpR. This would account for the absence of Ebp pili in EbpR containing strains grown in pilus-inducing conditions [66].

*E. faecalis* is known to aggregate platelets [57] a phenotype mediated, at least in part, by the Ebp pilus [58]. When comparing the ability of DBS01 and E99 to bind human platelets, DBS01 was found to adhere to platelets significantly (∼5 fold) better than E99 (Fig. 6). This ability to bind platelets is frequently implicated in promoting infective endocarditis [70], [71]. When the heart valves become damaged, platelet aggregation on the damaged tissue can serve as binding foci for circulating bacteria. In animal studies, these vegetations cause the further accumulation of platelets and bacteria onto the infected surface, a condition that may lead to heart failure or death [72].

PerA influenced the expression of a number of genes involved in amino acid metabolism. The majority of these genes comprise the ADI pathway (*arcABCRD*) in *E. faecalis*. The ADI pathway is used by *E. faecalis* to produce ATP via arginine fermentation [73], [74]. Expression of *arcABCRD* is tightly controlled as the ADI promoter region contains multiple binding sites for transcriptional regulators and catabolite repression elements [49]. Riboulet-Bisson et al. [50] recently identified an *Ers* (enterococcal regulator of survival) binding site upstream of the *arcA* gene, and suggested an activator role for this protein. In the current work, microarray analysis revealed that the ADI pathway is highly induced in DBS01, which is suggestive of PerA repression of these genes. Of interest is the increase in *arcABCRD* gene expression concomitant with the induction of the *ebp* locus in DBS01 (Fig. 3). During an infection, it is possible that these coordinately PerA-regulated genes perform a related function. In the presence of host proteins or amino acids, the de-repression of the *arcABCRD* operon would permit the transport and degradation of liberated arginine. In this scenario arginine fermentation may provide energy for biofilm formation during pathogenesis. The biofilms could then serve to increase bacterial persistence inside the host and further the invasion of nutrient-rich host tissue. Furthermore, the PerA regulon comprises genes encoding a putative peptide ABC transport system (Fig. 2). These peptide transport systems provide nutrients to the cell by internalizing amino acids and short peptides, and are often critical for the survival of auxotrophic lactic acid bacteria [32]. Zhu et al. [62] found that clinical isolates of *Staphylococcus aureus* selectively extracted arginine from growth media during biofilm formation. Chaussee et al. [75] found that in *Streptococcus pyogenes* the expression of virulence factors is coordinately regulated with amino acid catabolism. In this work, we show that PerA regulates genes involved in amino acid catabolism and biofilm formation, which further suggests a regulatory, if not functional, correlation between amino acid degradation and biofilm formation. While intriguing, the correlation between arginine metabolism and biofilm formation in *E. faecalis* remains to be studied.

Bicarbonate production is important for maintaining pH homeostasis in the small intestine, as it neutralizes acid in the intestinal lumen and prevents damage to the adherent mucus layer [76], [77]. Many pathogens use the presence of bicarbonate as an environmental signal to coordinate the expression of virulence traits and frequently AraC-type regulators are involved [46], [47], [78]. Bourgogne et al. have shown that the transcription of the *E. faecalis* OG1RF *ebp* locus is enhanced in the presence of bicarbonate, yet the regulatory cascade linking bicarbonate to *ebp* expression is unclear. In E99, PerA appears to be a repressor of *ebpABC* expression (Figs. 1B and 3). In the presence of bicarbonate *perA* was down-regulated concomitant with an induction of *ebpR-ebpABC* and *bps* expression (Fig. 4). This suggests that in E99, PerA may be part of the regulatory cascade controlling *ebp* expression in response to bicarbonate whereby the production of bicarbonate in the intestine causes a down-regulation of *perA*, which leads to the production of the Ebp pilus. In this scenario, the sensing of environmental bicarbonate ultimately stimulates the production of an adhesin that could aid in colonization of the intestine.

From our data we are unable to determine if PerA directly responds to bicarbonate or if it is influenced by other regulatory mechanisms that either detect bicarbonate or are influenced by the slight change in pH introduced by bicarbonate addition. AraC-type regulators are comprised of a conserved C-terminal DNA-binding domain and a N-terminal domain important for ligand binding. Comparisons of the PerA sequence to other AraC-type regulators that are known to detect bicarbonate (*C. rodentium* RegA and *V. cholerae* ToxT) reveal that PerA exhibits C-terminus similarity, yet virtually no N-terminus sequence similarity exists (data not shown). Furthermore, we have previously shown that the PerA N-terminus contains no similarities with other AraC-type regulators [30]. It is possible that PerA senses bicarbonate using a unique bicarbonate-binding motif, however it is also possible that other regulators that sense bicarbonate may control *perA* expression. In regards to the latter possibility, *E. coli* MarA and SoxS are AraC-type regulators known to regulate transcription without directly detecting a ligand [79], [80].

PerA also appears to influence the expression of a number of housekeeping genes. Perhaps most notably is the down-regulation of genes in DBS01 involved in the basic metabolism of the cell, concomitant with an induction of genes responsible for biofilm formation and attachment to host cells (Fig. 1B). It is possible that at the site of infection E99 uses PerA as a global dual-regulator to orchestrate the down-regulation of many housekeeping genes non-essential to pathogenicity while inducing genes responsible for colonization and infection of the host.

We have previously shown that PerA contributes to *E. faecalis* survival in the macrophage [30]. However, finding the PerA-regulated genes that coordinate macrophage survival using our current strategy has, thus far, proven inconclusive. We are keen to realize the harsh phagosomal environment encountered by *E. faecalis* during phagocytosis is almost certainly drastically different than the conditions in this study. Though studies seeking to determine the *E. faecalis* intracellular survival strategy have increased our understanding of the challenges faced upon phagocytosis, the whole-genome transcriptional response used by *E. faecalis* during macrophage survival has yet to be revealed. This information would not only yield a better understanding of the phagosomal landscape during *E. faecalis* infection, but it would also illuminate the *E. faecalis* macrophage survival strategy. During intracellular survival, it is possible that basal (or perhaps enhanced) expression of *perA* influences the transcription of hypothetical function genes, thus impacting persistence within the macrophage.

In the current study we used whole-genome *E. faecalis* V583 microarrays to determine the PerA regulon in E99. Though we used qRT-PCR to interrogate PAI genes in E99 that are missing from V583, we realize there could be other genes present in E99 yet absent from the V583 microarray. E99 contains a large, conjugative plasmid (pBEE99) comprised of genes that confer a high biofilm phenotype and increased ultraviolet radiation resistance [81]. Additionally pBEE99 contains genes putatively encoding an aggregation substance and a two-component bacteriocin [81]. Under the conditions tested PerA did not regulate either the PAI genes or the bee locus. However, the expression of the remaining pBEE99 genes in DBS01 remains to be determined. Furthermore, since the E99 genome has yet to be sequenced, this strain could possess unknown loci that are potentially regulated by PerA and contribute to virulence.

In conclusion, our data suggests that PerA is a global transcriptional regulator that coordinately controls genes important for pathogenicity. We can now propose a mechanism of how E99 achieves pathogenicity by using PerA as part of a regulatory network controlling expression of virulence genes. When appropriate environmental signals are sensed (quite possibly the presence of bicarbonate), the cell quickly and efficiently creates a rapid physiological change by down-regulating one gene: *perA*. In response to the environmental signal, the reduced levels of PerA would alleviate repression of genes important for biofilm formation and colonization of host tissues. Concurrently, metabolic and substrate transport pathways critical for cell nutrition are induced while unnecessary housekeeping genes are repressed, thus ensuring the cell has the proper nutrients for pathogenicity.

## Materials and Methods

### Bacterial strains, media, and reagents

The strains used in this study were *E. faecalis* E99 [34] and an isogenic Δ*perA::ermR* mutant (DBS01) [30]. The mutant DBS01 was complemented in *trans* as previously described [30]. The strains were routinely cultured in Todd-Hewitt broth (THB) containing 1% glucose or THB+1% glucose supplemented with 100 mM sodium bicarbonate when appropriate. Antibiotics used for selection included kanamycin (25 µg/ml) and erythromycin (50 µg/ml) (Sigma Chemical, St. Louis, MO). Growth was monitored as absorbance at 600 nm using a Beckman-Coulter DU800 spectrophotometer.

### RNA isolation and Microarray analysis

RNA extraction and microarray analysis proceeded as previously described [82] with a few modifications. Briefly, strains E99 and DBS01 were grown at 37°C overnight in THB+1% glucose in appropriate antibiotics. The bacteria were diluted 1∶10,000 into fresh, pre-warmed medium and incubated at 37°C. At predetermined optical densities (600 nm; 0.05 for mid-exponential, 0.5 for late-exponential, and 1.0 for stationary phase) cells were sampled directly into ice-cold RNA*later* (Ambion, Foster City, CA). Total RNA was extracted using Qiagen RNeasy Minikits (Valencia, CA) with optional on-column DNase treatment steps according to the manufacturer's specifications. RNA integrity was checked by gel electrophoresis and stored in 2 volumes of ethanol at −80°C. cDNA was generated by first strand synthesis using Superscript II (Invitrogen, Carlsbad, CA) and random hexamers according to the manufacturer's specifications. Fragmentation and biotinylation of cDNA proceeded according to the Affymetrix prokaryotic labeling protocol using the ENZO Kit from Roche Diagnostics (Indianapolis, IN). Biotinylated cDNA was hybridized to custom Affymetrix GeneChips for 16 h at 45°C. The custom microarrays used in this study contained probes for several prokaryotic genomes including *Enterococcus faecalis* V583 (GEO Accession number: GPL6702). Affymetrix protocol ProkGE_WS2v2-450 was used to stain the hybridized arrays. Following scanning, raw data files (.cel) were analyzed using RMA processing with quartile normalization [83]. Biological and technical replicates were averaged, and genes were considered to be significantly induced or repressed if the DBS01 : E99 expression ratio was greater than twofold [84]. Heatmaps were generated using DecisionSite for Functional Genomics (Spotfire, Somerville, MA). The microarray data has been deposited at GEO (GEO accession number, GSE31538). All data are MIAME compliant.

### qRT-PCR

Transcript levels were confirmed by qRT-PCR using RNA extracted from cells harvested during mid-exponential, late-exponential, and stationary phase. The primers listed in Table 1 were designed using Primer Express software provided with the ABI Prism 7000 sequence detector (Applied Biosystems, Foster City, CA). Amplicon lengths were 100 bp. Quantification of 16 S rRNA levels was used as an internal control and to normalize RNA. Amplification was detected using SYBR Green PCR Master Mix (Applied Biosystems) with automatic calculation of threshold value (C_T_). The fold changes in gene expression were determined by comparing mRNA abundance in DBS01 to that in E99 as previously described [85]. Analysis was repeated in triplicate on two biological replicates for each time point. Replicates were averaged and the results are presented in Table 2.

**Table 1:** qRT-PCR primers used in this study.

Primer	Sequence (5′ - 3′)
*arcA*-F	AAGCCAATATTCGCAGCGAA
*arcA*-R	AATGCCTGCAATCGCTTTTT
*arcB*-F	TTTGACGGGATTGAGTTCCG
*arcB*-R	TGCCATTGATCCGTTAAACCA
*arcC*-F	ATGATGCTAGCGCACATGCA
*arcC*-R	GCCATGTGAAACAATCAACCG
*arcR*-F	TCCGAGAATCGGACTGTTTCA
*arcR*-R	AACGCTCAAACAGTTTAACTGGC
*ebpA*-F	ACCGCGGATGAAAGCTATCA
*ebpA*-R	CCAGGAACTGCTAATTCACGG
*ebpB*-F	CGTACAGGCGGCAAGTCTTT
*ebpB*-R	AGGTATTCCCCCGCTTGATT
*ebpC*-F	GAATTTTACGAGCAACGAGCG
*ebpC*-R	TCGGTGGTTCCTTGAGCAAC
*bps*-F	CATTTCAGGCCATCGTGGTC
*bps*-R	GCGTCTTCCCATTGACTTCG
16S-F	AGCCGGAATCGCTAGTAATCG
16S-R	TCGGGTGTTACAAACTCTCGTG

**Table 2:** Members of the PerA regulon confirmed by qRT-PCR.

		Fold-Change*		
Gene	Product	0.05	0.5	1.0
*ebpA*	von Willebrand factor	7.8 (0.07)	30.0 (0.05)	21.1 (0.02)
*ebpB*	Cell wall surface protein	14.0 (0.03)	30.0 (0.03)	19.7 (0.02)
*ebpC*	Cell wall surface protein	14.0 (0.1)	27.9 (0.06)	19.7 (0.1)
*bps*	Sortase	2.6 (0.05)	2.8 (0.06)	2.0 (0.01)
*arcA*	Arginine deiminase	30.0 (0.03)	274.4 (0.1)	9.8 (0.03)
*arcB*	Ornithine carbamoyltransferase	24.3 (0.01)	181.0 (0.3)	13.9 (0.04)
*arcC*	Carbamate kinase	8.0 (0.1)	73.5 (0.2)	19.7 (0.05)
*arcR*	Transcriptional regulator Crp/Fnr	4.3 (0.1)	64.0 (0.1)	18.4 (0.08)

* Change in DBS01 gene expression (DBS01 : E99) at OD600 = 0.05, 0.5 and 1.0.

Experiments were repeated twice, with 3 replicates for each gene per assay. Mean values shown (standard error in parenthesis).

### Assessment of Platelet Binding

The ability of *E. faecalis* cultures to bind human platelets was assessed as previously described [55]. Briefly, human platelets were washed, fixed and immobilized on poly-L-lysine-coated 22-mm-diameter tissue culture wells at a concentration of 1×10^8^ platelets per well. Following 30 min incubation at 37°C, unbound platelets were removed by aspiration. The remaining bound platelets were subsequently incubated in a 1% casein solution for 1 h at 37°C to reduce non-specific adherence. Following removal of the blocking solution, each well was inoculated with 1×10^8^ of *E. faecalis* E99, DBS01, or DBS01 (pGT101) suspended in PBS and further incubated with gentle rocking. After 1 h unbound bacteria were removed by washing each well twice with PBS and the bound bacteria were collected by scraping and resuspending them in PBS. The number of bacteria bound to platelets was determined by plating suspensions on THB supplemented with appropriate antibiotics. Binding was expressed as a percentage of the inoculum. Platelet binding assays were performed three times, each assay replicated in triplicate (n = 9) using blood from multiple, healthy volunteers. Differences in platelet binding efficiencies were determined using an unpaired *t*-test, as shown in Fig. 6.

### Ethics Statement

This study was performed under the supervision and approval of the Institutional Review Board at the University of Oklahoma. The platelets used in this study were purchased from Bioreclamation (Long Island, NY) and obtained from a blood bank supplied by healthy volunteers.

## Supporting Information

Table S1 The gene, locus tag, and annotated gene product for the *E. faecalis* E99 PerA regulon.
(XLSX)
